# Trans-Corneal Subretinal Injection in Mice and Its Effect on the Function and Morphology of the Retina

**DOI:** 10.1371/journal.pone.0136523

**Published:** 2015-08-28

**Authors:** Yan Qi, Xufeng Dai, Hua Zhang, Ying He, Yangyang Zhang, Juanjuan Han, Ping Zhu, Yuxin Zhang, Qinxiang Zheng, Xia Li, Chen Zhao, Jijing Pang

**Affiliations:** 1 School of Ophthalmology & Optometry, The Eye Hospital, Wenzhou Medical University, Wenzhou, Zhejiang, P. R. China; 2 Fenyang College of Shanxi Medical University, Fenyang, Shanxi, P. R. China; 3 Department of Ophthalmology, College of Medicine, University of Florida, Gainesville, Florida, United States of America; 4 Department of Ophthalmology, First Affiliated Hospital, Nanjing Medical University, Nanjing, Jiangsu, P. R. China; 5 Department of Ophthalmology, First Affiliated Hospital of Guangxi Medical University, Nanning, Guangxi, P. R. China; Cedars-Sinai Medical Center; UCLA School of Medicine, UNITED STATES

## Abstract

**Purpose:**

To introduce a practical method of subretinal injection in mice and evaluate injection-induced retinal detachment (RD) and damage using a dynamic imaging system, electrophysiology, and histology.

**Methods:**

After full dilation of a 2-month-old C57BL/6J mouse pupil, the cornea near the limbus was punctured with a 30 ½-gague disposable beveled needle. A 33 ½-gauge blunt needle was inserted through the corneal perforation into the anterior chamber, avoiding the lens before going deeper into the vitreous cavity, and penetrating the inner retina to reach the subretinal space. The mice were divided into four groups: in group 1, about 80–100% of the retina was filled with subretinally injected solution; in group 2, approximately 50–70% of the retina was filled with injected solution; in group 3, the procedures were stopped before solution injection; and non-injected eyes were used as the negative control in group 4. An optical coherence tomography (OCT) imaging system was used to monitor retinal reattachment during the first three days following the injections. Histological and functional changes were examined by light microscopy and electroretinography (ERG) at five weeks post-injection.

**Results:**

After a short-term training, a 70% success rate with 50% or more coverage (i.e., retinal blebs occupied 50% or more retinal area and filled with the injected solution) with minimal injection-related damages can be achieved. Bleb formation was associated with retinal detachment (RD) between the neuroretina and the retinal pigment epithelium (RPE) layer. Partial RD could be observed at post-injection day 1, and by day 2 most of the retina had reattached. At 5 weeks post-injection, compared to uninjected control group 4, the b-wave amplitudes of ERG decreased 22% in group 1, 16% in group 2, and 7% in group 3; the b-wave amplitudes were statistically different between the uninjected group and the groups with either 50–70% or 80–100% coverage. The subretinal injection-induced RD reattached and became stable at five weeks post-injection, although some photoreceptor damage could still be observed in and around the injection sites, especially in 80–100% coverage group.

**Conclusions:**

Trans-corneal subretinal injection is effective and practical, although subretinal injection-related damages can cause some morphological and functional loss.

## Introduction

To date, over 60 genes have been linked to inherited forms of retinitis pigmentosa (RP) (Retnet: www.sph.uth.tmc.edu/retnet/). In recent decades, more and more mouse models of congenital retinal diseases have been found or created and are used to test the efficacy in preclinical trials of retinal gene therapy [1]. A variety of gene therapy studies has shown the rescue of retinal function [2–7]. These studies utilized subretinal injections, which is the most effective way to transfect RPE and/or photoreceptor cells, since a great majority of the mutant genes associated with retinal degeneration is expressed in rods, cones, or RPE cells, which are close to the subretinal space. Furthermore, the subretinal space has a relatively high degree of immune privilege and is thus considered an ideal route for the delivery of vectors [5,8]. Tight junctions that form the blood-retina barrier separate the subretinal space from blood supply, thereby protecting it from immune-mediated damage that could lead to inflammatory processes and prevent vector-mediated transgene expression [8].

It is known that subretinal injection can lead to RD, which is a very common clinical disease and can result in serious vision loss. Recently published results for 15 patients from the current National Eye Institute (NEI)-sponsored Leber congenital amaurosis type 2 (LCA2) clinical trial (NCT00481546) showed that single or multiple subretinal adeno-associated viral (AAV) vector injections resulting in vector blebs in paramacular and more peripheral retinal areas uniformly led to robust quantitative improvements in light sensitivity, visual acuity, pupillary light response, and mobility performance [9–11]. On the other hand, vector blebs that detached the fovea resulted in little or no vision-gain for the patient and in some, a slight loss in visual acuity [11]. Whether or not the RD caused by subretinal injections could influence retinal function in mice is unclear as mice have no macula or fovea.

**Fig 1:**
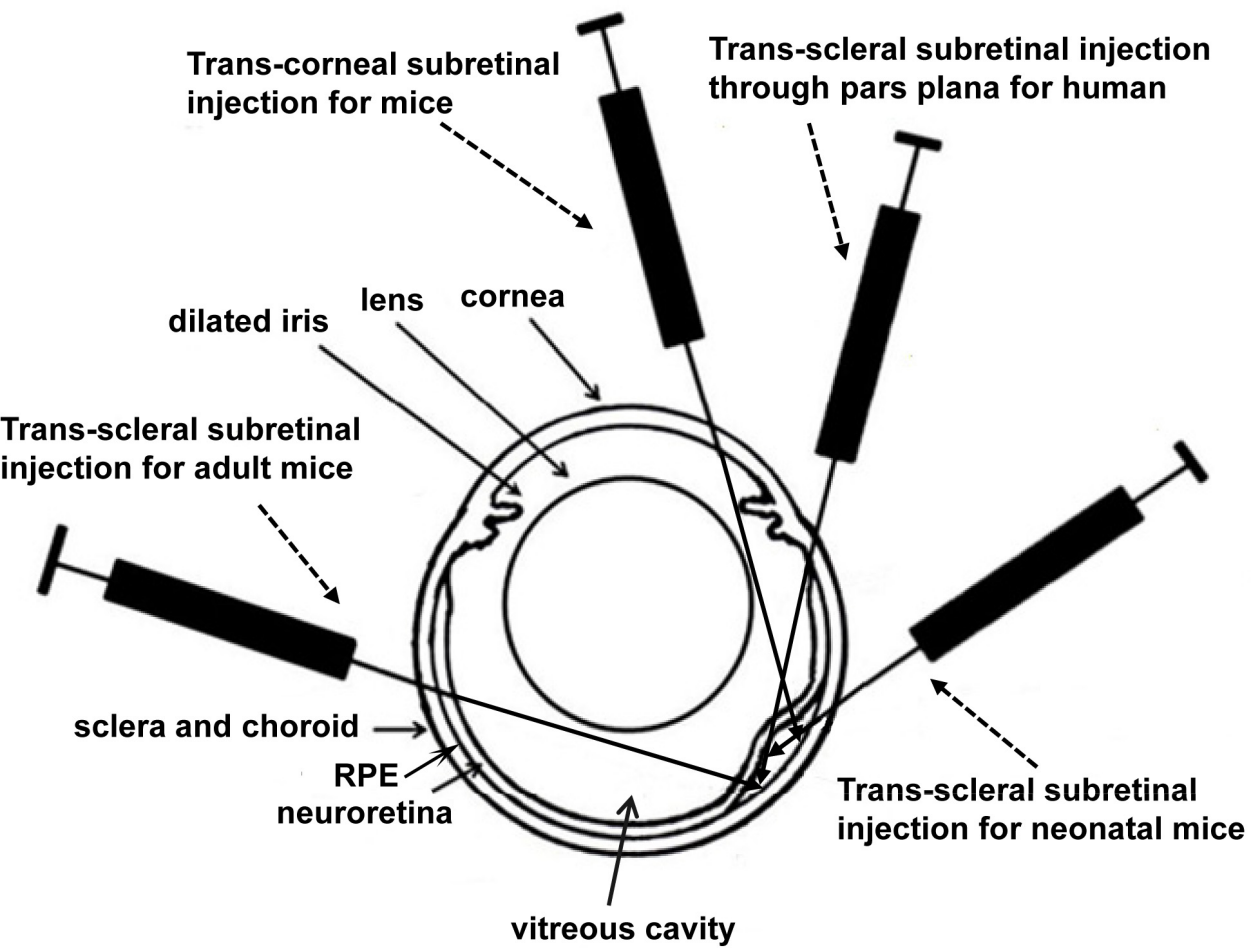
Diagram of different routes for subretinal injections. This diagram showed the routes of trans-corneal and trans-scleral subretinal injections including the different routes to adult and neonatal mice.

There are different routes for subretinal injection [12,13]. Trans-scleral subretinal injection through pars plana, which locates in the anterior part of the sclera close to limbus, is usually used for humans [9–11] or large animals with relatively small lens. This can ultimately avoid the injection-related corneal and retinal injuries. Trans-scleral subretinal injection through the middle or posterior part of the sclera is also used for rodents with big lens, and is unique for neonatal rodents before they open their eyes. For neonatal mice, trans-corneal subretinal injection cannot avoid damages to cornea, iris, lens and retina because of the difficulty in pupil dilation and that these tissues are still in developing stage. As a result, trans-scleral subretinal injection has to be used in neonatal mice although the injection-related damages are extensive [14,15]; in order to minimize the damages, the injection needle has to stop in the subretinal space of the initial side but not the other side of the eyeball (Fig 1), which makes reflux a common phenomenon. This method usually can make less than 50% of RD/transduction coverage but massive damages in the cornea, lens and retina [14–16]. That is why researchers usually like to do as much as they can to avoid using subretinal injection to neonatal mice in a rescue study, even though an early intervention is beneficial to the degenerating retina [5,8].

Trans-scleral subretinal injection through the middle or posterior part of the sclera is also widely used for adult mice, in which the inserting needle may either penetrate one side of the eyeball to reach the subretinal space of the other side, or stop in the subretinal space of the same side of the eyeball as the method used in neonatal mice; although it is easy to learn and practise for untrained beginners, this method has several major drawbacks: it usually detaches less than half of the retina with leakage and/or cause more retinal /lens damage [12,17,18]. In the past 10 years, more and more researchers have been using trans-corneal subretinal injection to treat mouse models of retinal diseases. Trans-corneal subretinal injection is usually used in postnatal day 14 (P14) or older rodents with relatively big lenses. Since it has the unique advantage of detaching the whole retina [19], trans-corneal subretinal injection of vector encoding a therapeutic gene has been used in many mouse-models of inherited retinal diseases to correct gene defects [2–5,7,20]. The difference of these subretinal injection methods can been seen in Fig 1. Although this method has been described and has shown reliability in rats [21], no study has shown evidence on the duration of retinal reattachment using a non-invasive measurement in mice. Additionally, it is unclear whether there is any permanent functional loss if nearly 100% of the retina detaches following trans-corneal subretinal injection in mice.

In the present study, normal saline with sodium fluorescein as a dye was injected into the subretinal space through the cornea. Retinal reattachment was observed through optical coherence tomography (OCT) on post-injection days 1, 2, and 3. Retinal function and structure were assessed by electroretinographic (ERG) measurements and paraffin sections at 5 weeks post-injection.

## Materials and Methods

### Animals

C57BL/6J mice were purchased from Shanghai Silaike Experimental Animal LLC (Shanghai, China) and were maintained and bred at the Wenzhou Medical University animal facilities under a 12-hour light / 12-hour dark illumination cycle with free access to food and water. All experiments were conducted in accordance with the Association for Research in Vision and Ophthalmology (ARVO) Statement for the Use of Animals in Ophthalmic and Vision Research and approved by the Wenzhou Medical University Institutional Animal Care and Use Committee. Experimental mice were divided into four groups: in group 1, subretinally injected solution covered approximately 80–100% of the retina; in group 2, approximately 50–70% of the retina was covered; in group 3, the procedures were stopped before solution injection (pseudo-injection group) and only those eyes with entire automatic RD were used for further evaluation [19]. Age-matched non-injected eyes were used as the negative control (group 4). To differentiate the blebs filled and unfilled with injected solution, 0.1% sodium fluorescein was added to the normal saline as a green dye. Only those blebs with green color underneath were counted as RD filled with injected solution. The subretinal injection method has been briefly described in our previous studies [3–7]. The detailed protocol is stated below, including the pre-surgical preparations, post-injection care and injection procedures.

#### Preparation on the Day before Injection

During late afternoon, a drop of 1% atropine (Alcon Laboratories Inc., Fort Worth, TX, USA) was applied into each eye followed by another drop after an hour. For topical drop applications, surgeons should ensure the drop stays in the eye for at least 10 seconds (s). If the drop rolls off the eye before 10 s, another drop should be applied.

#### Preparation on the Day of Injection

1. Five hours before the injection (based on the planned injection time), one drop of 1% atropine was applied to each eye for a total of 4 times, approximately 1 hour apart.

2. Two hours before the injection (based on the planned injection time), one drop of 2.5% phenylephrine hydrochloride (Akorn Inc., Decatur, IL, USA) was applied to each eye every 30 min, for a total of 5 times. Mice were anesthetized by an intraperitoneal injection of a mixture of ketamine (72 mg/kg) and xylazine (4 mg/kg) as previously described [22], followed by one more drop of 2.5% phenylephrine into each eye.

4. The animal was positioned on a table with a movable flat card as the surgery platform. A 1-mL insulin syringe was taped to the card to be used as a pillow to elevate the mouse head.

#### Injection Procedures

**Fig 2:**
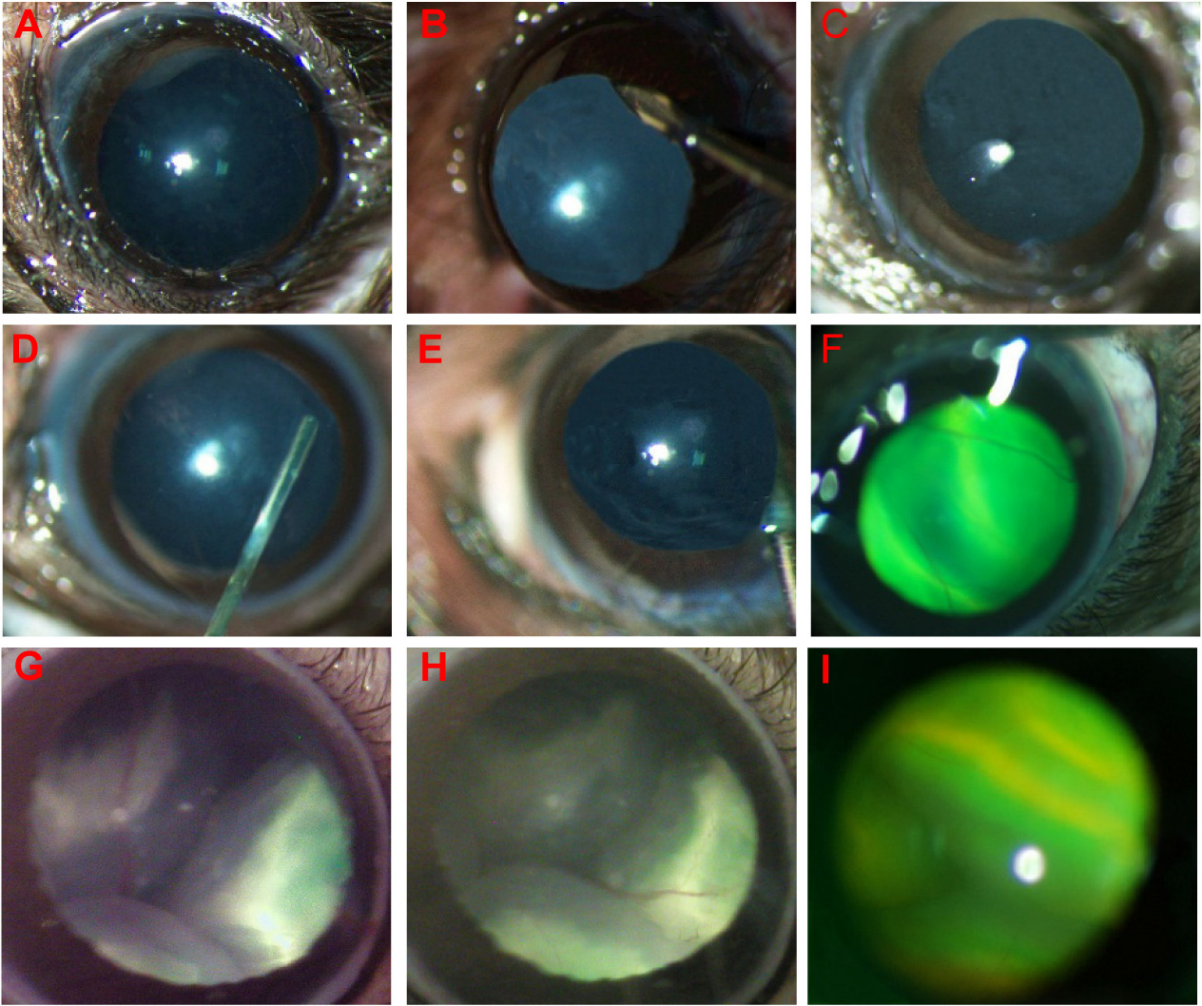
Steps of trans-corneal subretinal injection in mice. A. Fully dilated pupil before injection. B. Cornea punctured with a 30 ½-gauge disposable needle. C. A generated hole. D. A 33 ½-gauge blunt needle placed into the anterior chamber through the hole. E. The needle bypasses the lens and iris before reaching the subretinal space followed by injection with 1 μl normal saline containing sodium fluorescein. F. A successful subretinal injection with retinal blebs on which retinal blood vessels are seen. G. Less than 50% coverage: one of three blebs was filled with injected fluorescein sodium. H. 50–70% coverage: two of three blebs were filled with injected fluorescein sodium. I. 80–100% coverage: all blebs were filled with injected fluorescein sodium.

1. An additional drop of 2.5% phenylephrine was applied following anesthesia (Fig 2A).

2. One drop of 0.5% proparacaine hydrochloride (Alcon Laboratories Inc., Fort Worth, TX, USA) should be applied for local anesthesia if necessary.

3. One drop of 2.5% hydroxypropyl methylcellulose (Akorn Inc., Lake Forest, IL, USA) or sodium hyaluronate (Abbott Medical Optics Inc., Chicago, IL, USA) was placed on the cornea to protect the cornea and for better visualization during the procedures.

4. Under direct visualization of a dissecting microscope, an incision was made in the cornea near the limbus but within pupil range using a 30 ½-gague disposable beveled needle (Becton Dickinson & Company, Franklin Lakes, NJ, USA). At the start of penetration, the needle was placed vertical to the corneal surface, and the needle orientation was then changed to be parallel to the anterior surface of the lens just before penetrating the cornea. This avoids possible injuries to the lens and iris and reduces the extent of automatic retinal detachment due to rapid outflow of aqueous humor (Fig 2B and 2C). Next, a 33 ½-gauge blunt needle (Hamilton Company, Reno, NV, USA) with a Hamilton microsyringe was inserted into the eye through the corneal perforation into the anterior chamber (Fig 2D), avoiding the iris and lens to go deeper into the vitreous cavity and was stopped before the retinal inner surface (Fig 2E).

5. The microscope was refocused by an assistant, and an area with a lower number of blood vessels was selected before penetrating the inner retina to reach the subretinal space.

6. The plunger of the Hamilton syringe was slowly pushed by the assistant, taking up to 45 s to deliver 1.0 μL normal saline with 0.1% sodium fluorescein (Alcon Laboratories Inc., Fort Worth, TX, USA) into the subretinal space.

7. The needle was slowly removed and an additional drop of hydroxypropyl methylcellulose or sodium hyaluronate was applied before checking the percentage of retina with subretinal fluorescein and for any injection-related damage or bleeding immediately after the procedure (Fig 2F).

8. The injection procedure should be completed in the 2–3 minutes following anesthesia administration to avoid anesthesia-related cataract.

9. Following all injections, 1% atropine eye-drops and neomycin/polymyxin B/dexamethasone ophthalmic ointment (Alcon Laboratories Inc., Fort Worth, TX, USA) were applied daily for three days and then every two days for a total of one week.

### OCT Imaging

On post-injection days 1, 2, and 3, pupils of both injected and non-injected eyes were dilated with 1% atropine and 2.5% phenylephrine hydrochloride. Mice were then anesthetized as described in the subretinal injection section. One drop of 2.5% hydroxypropyl methylcellulose was administrated to the eyes before examination. OCT was performed using Micron Ⅳ retinal imaging system (Phoenix Research Labs, Pleasanton, NJ, USA). OCT and fundus photographic camera system were operated at the same time during examination. In order to obtain the fundus image, the animal was positioned so that the cornea touched the camera lens. Retina was subjected to a linear horizontal scanning referred to as the fundus image. OCT was adjusted to make clear images and the images were acquired after noise reduction of the average.

### ERG Recording

At 5 weeks after subretinal injection, a RETI-port system with a custom-built Ganzfeld dome (Q450SC UV; Roland Consult, Wiesbaden, Germany) was used for ERG recording to assess the effect of the subretinal injections on retinal function. All procedures were performed in a dark room under dim red light illumination (>650 nm). The animals were anesthetized as described in the subretinal injection section after overnight dark-adaptation. The pupils were dilated with a drop of the compound tropicamide. One drop of 0.5% proparacaine hydrochloride was applied as topical anesthesia before the electrodes were attached. ERGs from both eyes were recorded simultaneously. For recording the ERGs, a pair of gold loop electrodes was placed on the surface of both eyes, a reference electrode was inserted subcutaneously into the mouse head in the area between the ears, and a ground electrode was inserted into the tail of the animal. In order to enhance electrical conductibility, one drop of 0.5% sodium carboxymethyl cellulose (Allergan Inc., Irvine, CA, USA) was placed on the cornea. The animals were superposed on a thermal platform that was maintained at 37°C. The head was held on the platform to prevent the displacement of the mouse from movement during the procedure. Full-field scotopic ERGs of both eyes were recorded at -1.85 and 0 log cd s/m^2^ intensity. For light-adapted ERGs, the animals were put under a background light of 30 cd s/m^2^ for 10 minutes before recording. Photopic ERGs were measured at 0.65 log cd s/m^2^ intensity.

### Morphological and Histological Analysis

The mice were sacrificed by cervical dislocation after ERG testing. Eyes were enucleated and fixed with 10% formaldehyde in phosphate-buffered saline for histology. Eyecup was prepared by the immediate removal of cornea and maintaining in fixer overnight, followed by iris and lens removal. Next, the fixed eyecups were dehydrated by automatic tissue hydroextractor (KD Inc., Zhejiang, China). Following dehydration, the eyecups were embedded in paraffin for one day and sectioned at 5 μm. The sections were stained with hematoxylin and eosin (H&E) before being photographed with a Zeiss bright-field microscope (Axio Imager Z1; Carl Zeiss Meditec, Oberkochen, Germany). Images of the retinal structure were obtained for analysis.

### Statistical Analysis

SPSS 21.0 (IBM Corporation, Armonk, NY) was used for statistical analysis. ERG data are presented as mean ± SD. Statistical analysis was performed with Kruskal-Wallis Rank Sum Test. A P value of < 0.05 was considered significant.

## Results

### Subretinal Injection

The retinal blood vessels could be visualized on the retinal blebs with green dye underneath, suggesting that the injected solution with dye was in the subretinal space [17,21]. A successful subretinal injection should transfect no less than 50% of the retina with minimal injection-related complications [5,6]. After practice, 50–70% RD filled with injected solution can be achieved in about 40% of total injections, and 80–100% coverage in 30% of total injections. Only successfully injected mice were used for further evaluation. The other 30% of unsuccessful injections may result from either less than 50% retinal blebs filled with injected solution or severe injection-related complications, such as massive iris and retinal hemorrhage, severe cataract, massive corneal opacity, and/or cornea-iris adhesion.

### OCT Evaluation

**Fig 3:**
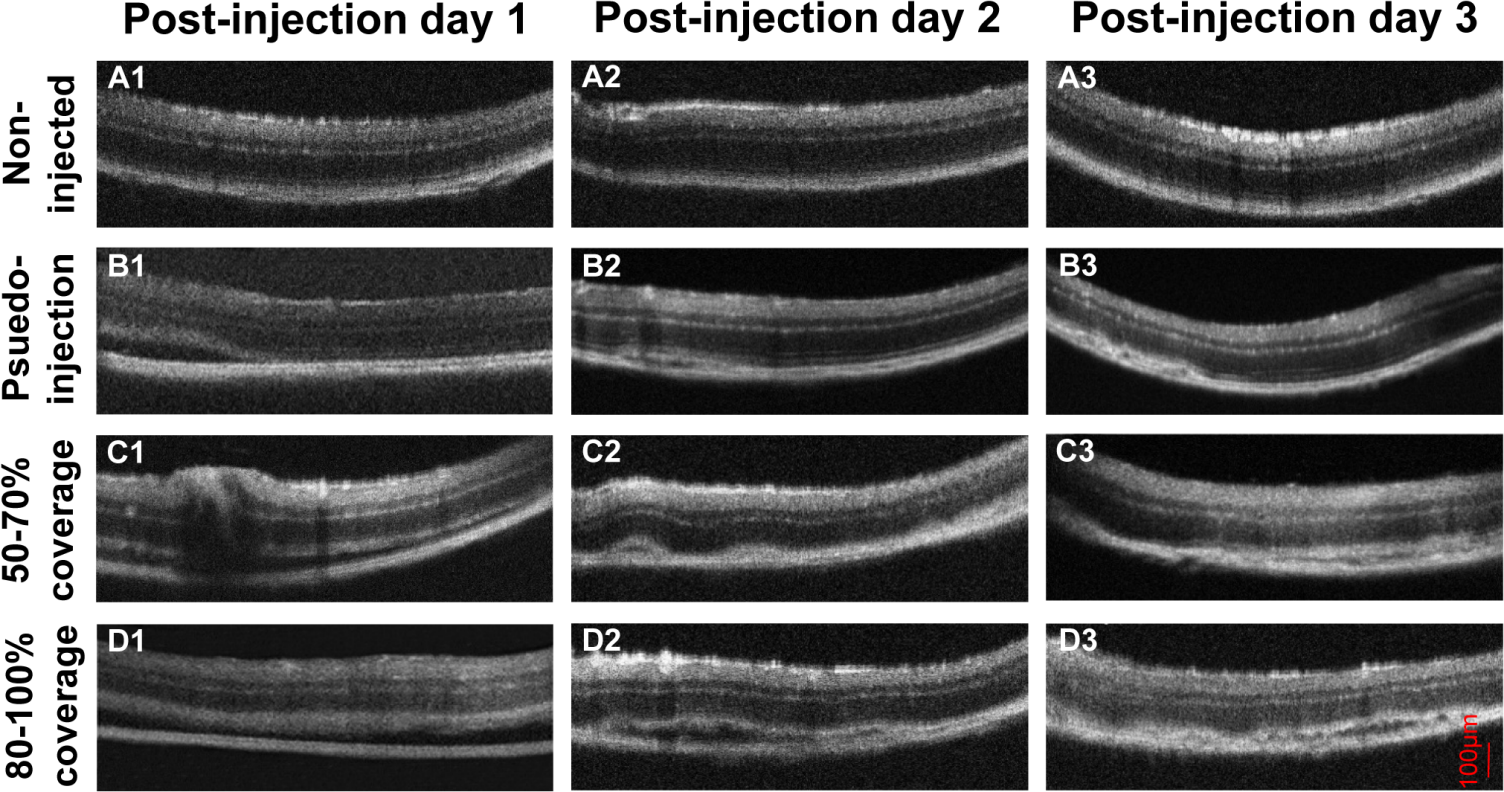
OCT images of mouse retinas. The OCT images of A1-A3 were from age-matched uninjected control C57BL/6J eyes. The OCT images showed retinal re-attachment at post-injection days 1 (B1), 2 (B2), and 3 (B3) in the pseudo-injection group; at post-injection days 1 (C1), 2 (C2), and 3 (C3) in the group with 50–70% coverage; and at post-injection days 1 (D1), 2 (D2), and 3 (D3) in the group with 80–100% coverage.

In most mice, retinal re-attachment occurred on post-injection day 1 or day 2. The retinas of age-matched C57BL/6J eyes without injection were intact and neuroretina tightly attached to retinal pigment epithelium (RPE) (Fig 3A1, 3A2 and 3A3). Compared with the non-injection group, the pseudo-injection group showed different results under our procedures: half of the eyes did not show RD at the time of the procedures and the other half showed up to 100% automatic RD immediately after subretinal injection, perhaps due to negative pressure by aqueous humor drain from the corneal perforation [19]. On post-injection day 1, most detached retinas were reattached, but some showed small fissures between the neuroretina and RPE layer, indicating minor RD (Fig 3B1); Majority of detached areas reattached on post-injection day 2, and the remaining fissure between the neuroretina and RPE layer became smaller and more limited (Fig 3B2). On post-injection day 3, a more complete retinal reattachment was seen and the neuroretina and RPE layer attached more tightly (Fig 3B3). In the group with 50–70% coverage, minor RD could be observed in limited area of the eyes at post-injection day 1 (Fig 3C1); on post-injection days 2 and 3, the retinal structure gradually returned to normal (Fig 3C2 and 3C3). In the group with 80–100% coverage, the neuro-retina and RPE layer were still separated slightly in many parts of the retina on post-injection day 1 (Fig 3D1); retinas re-attached dramatically on post-injection day 2, although very limited RD could still be observed occasionally (Fig 3D2); on post-injection day 3, a more complete retinal re-attachment was observed (Fig 3D3).

### Electroretinographic Analysis at 5 weeks Post-injection

**Fig 4:**
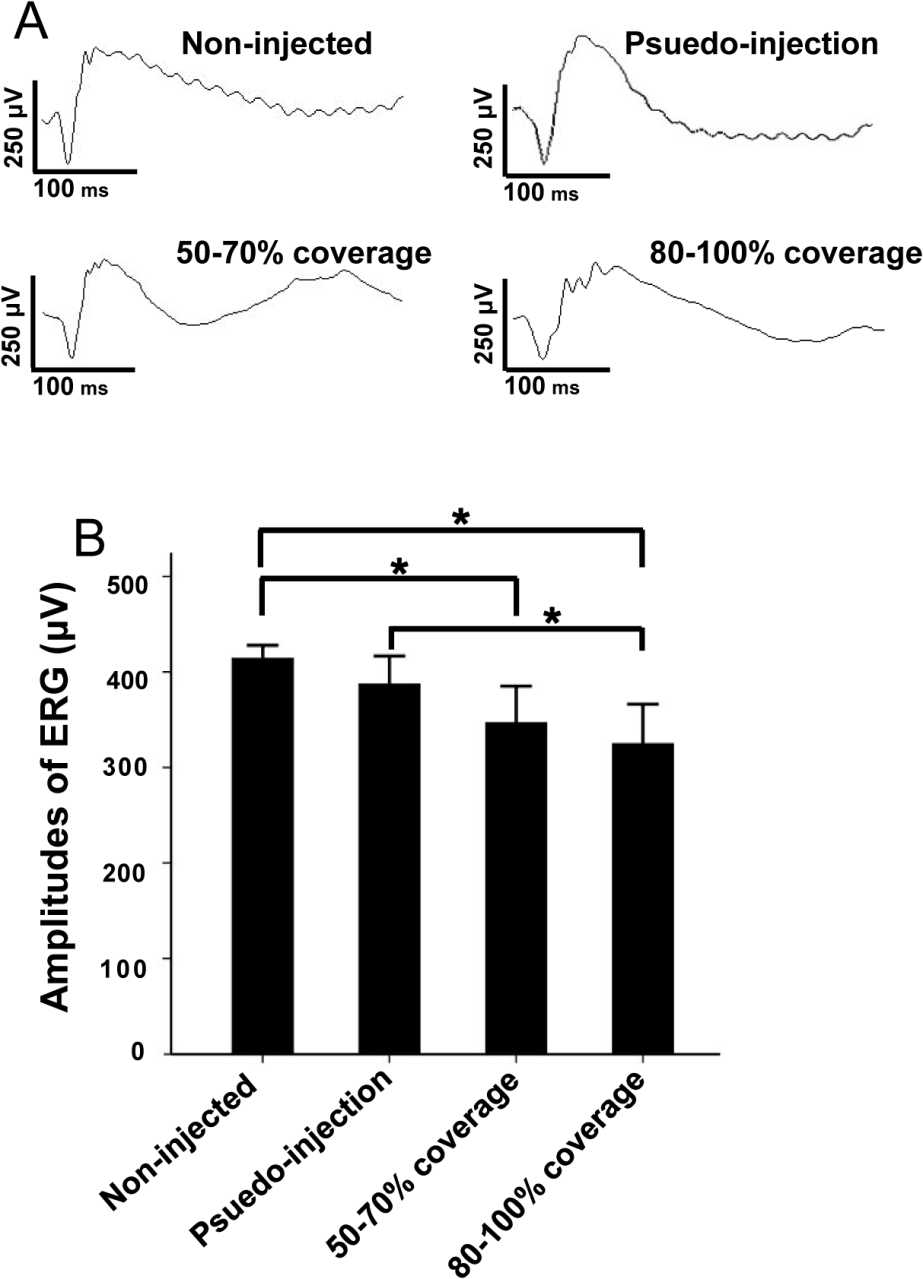
The b-wave amplitudes of standard combined ERG at 5 weeks post-injection. A. B-wave amplitudes of standard combined ERG signals from four groups of mice. B. Statistical analysis of the b-wave amplitudes. Rod and cone mixed responses were elicited at 0 log cd s/m^2^ intensity. N = 5 for each group. B-wave amplitudes of the non-injected group were higher than those from the other group. *P < 0.05.

At five-week after injection, the b-wave amplitude of standard combined ERG was 387.20 ± 32.88 μV in the pseudo-injection group, 346.80 ± 42.84 μV in the group with 50–70% coverage, and 324.80 ± 46.31 μV in the group with 80–100% coverage, while the b-wave amplitudes of eyes from the uninjected control group was 414.20 ± 15.32 μV (Fig 4A). Compared with those in the uninjected control group, the b-wave amplitudes of ERG declined 22% in the group with 80–100% coverage, 16% in the group with 50–70% coverage, and 7% in the pseudo-injection group (Fig 4B). There was no statistical difference in the b-wave amplitudes between the uninjected and pseudo-injection groups (p > 0.05). Meanwhile differences were statistically significant between the groups with either 50%-70% or 80%-100% coverage and the uninjected group (p < 0.05). No significant difference was observed in the b-wave amplitudes between the groups with 50–70% and 80–100% coverages (p > 0.05). Meanwhile differences were statistically different between the pseudo-injection group and the group with 80–100% coverage (p<0.05, Fig 4, Table 1).

**Table 1:** statistical analysis of the b-wave amplitudes.

Test	Ranks (n = 5)	statistics	P-value
**Control**	**0**		
**Psuedo-injection**	**+**		
**50–70% coverage**	**++**		
**80–100% coverage**	**+++**		
**Overall**		**χ** ^**2**^ **= 10.016**	**0.018**
**pseudo-injection vs. uninjected**		**U = 6.5**	**0.209**
**50–70% coverage vs. uninjected**		**U = 2.0**	**0.028**
**80–100% coverage vs. uninjected**		**U = 0.0**	**0.009**
**pseudo-injection vs. 50–70% coverage**		**U = 6.0**	**0.175**
**pseudo-injection vs. 80–100% coverage**		**U = 3.0**	**0.047**
**50–70% vs. 80–100% coverage**		**U = 10.0**	**0.602**

### Changes of Retinal Structure at 5 weeks Post-injection

**Fig 5:**
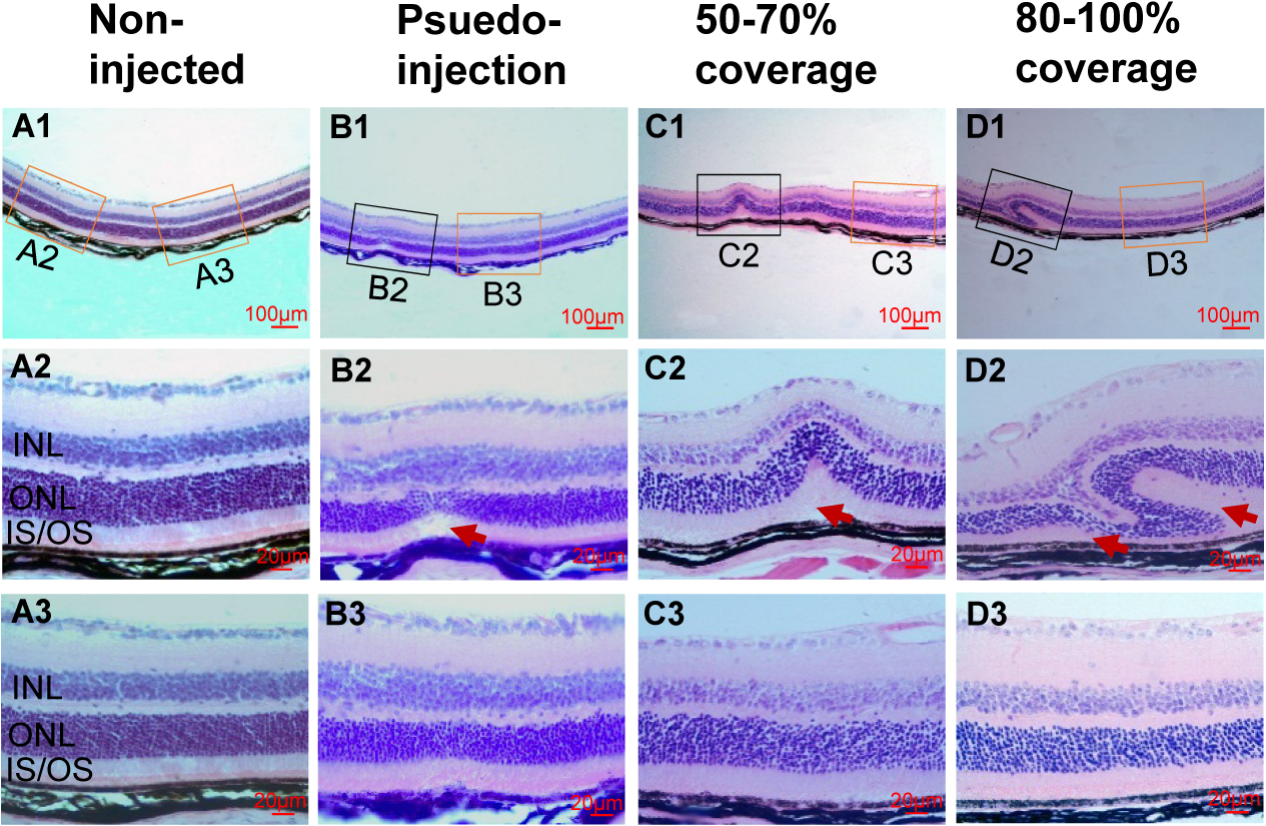
Histopathology of retinas by H&E staining from 4 groups of mice at 5 weeks post-injection. (A) Representative retinal images of H&E-stained retinal section from age-matched normal uninjected eyes (A1–A3; A2 and A3 are insets of A1 with yellow-frames). (B) Representative retinal image from the pseudo-injection group (B1–B3; B2 is inset of B1 with black-frame, B3 is inset of B1 with yellow-frame). (C) Representative retinal images from the group with 50–70% coverage (C1–C3; C2 is inset of C1 with black-frame, C3 is inset of C1 with yellow-frame). (D) Representative retinal images from the group with 80–100% coverage (D1-D3; D2 is inset of D1 with black-frame, D3 is inset of D1 with yellow-frame). The structure of retinas outside of injection sites were normal (B3, C3 and D3). ONL, outer nuclear layer; INL, inner nuclear layer; IS, inner segment; OS, outer segment; red arrow, injection-related damages of outer segments.

To investigate the change in retinal structure after injection, we used H&E staining of paraffin sections from each group to evaluate the retinal histology. The H&E-stained retinal sections from eyes enucleated at 5 weeks post-injection were examined by light microscopy. To find if the larger percentage of RD filled with injected solutions may result in irreversible pathological changes, we examined the different groups and found no major changes in the samples except in the area of injections. The detached neuroretina had flattened and reattached to the RPE in the area outside the injection site (Fig 5B3, 5C3 and 5D3). The abnormal changes in the area of injection were mainly malposition and damage of the photoreceptor cells.

In the non-injected group, the neuroretina and RPE layer were connected tightly, and the retinal structure was normal (Fig 5A). Only minor damage in the outer nuclear layer and outer segments could be observed around the injection site (Fig 5B1/black box, 5B2/arrow) in the pseudo-injection group. In the group with 50–70% coverage, malposition of the inner and outer nuclear layers was observed occasionally (Fig 5C1 and 5C2/arrow). In the group with 80–100% coverage, a major malposition and/or damage of the inner and outer nuclear layers were observed around the injection site (Fig 5D1 and 5D2); in the area close to the injection site, shortened outer segments were also observed occasionally (Fig 5D2/left arrow).

## Discussion

Subretinal injections provide feasibility for gene therapy or stem cell transplantation in the treatment of retinal degenerative diseases [16]. The subretinal space is located in the potential cavity between RPE and photoreceptor cells. Due to the close connection between the RPE cells, the monolayer RPE forms a blood retinal barrier, which controls the molecular exchange between the retina and choroid. RPE cells secrete immunosuppressive and anti-inflammatory molecules; these factors constitute the immune privilege of subretinal space, which is an important feature of the eye, making the subretinal injection a useful method for retinal gene therapy [8,23]. Subretinal injection is commonly used to treat rodent models of degenerative retinal diseases. Comparing other subretinal injection methods using more than 1 μl of solution but only detaching less area of retina [21], we can detach and transfect almost the entire retina at the end of this trans-corneal subretinal injection procedures. As described in a previous study [19], quick intraocular pressure drop by cornea-puncture can cause automatic RD and can recover in 1 day. Our method using the modified procedure can also cause automatic retinal detachment. An experienced injector can reduce the rate or extent/size of automatic RD by decreasing and slowing the cornea-puncture induced aqueous humor leakage (see details in methods), but cannot avoid the automatic RD in every case, and that’s why we designed a pseudo-injection group with the entire retina detached. But, that is also one of the advantages and reasons why we can use 1 μl solution to transfect/cover 100% retina because it is easier for vector solution to spread in the subretinal space of detached retina and a perfect injection can cause little leakage during injection, which is another reason why 1 μl solution can spread to the entire retina with our method. In fact, we found that a perfect injection using 2 or 3 μl solution without any leakage can cause bigger blebs and more injection-related irreversible damages, for example the retinal inner surface-lens adhesion that can block the reattachment of detached retina following injection and cause more photoreceptor death and ERG reduction (data not shown).

Although some failed injections could lead to no RD/bleb at all with this method, many injected eyes showed entire RD with blebs throughout the retina (Fig 2). In order to differentiate whether the bleb is filled with injected solution or not, we added the fluorescein green dye in the injecting solution. In that case, only eyes with retinal blebs that filled with injected green solution are counted, which is critical to gene therapy-mediated rescue project. The reason why we divided the injected eyes into three groups is that following injection procedures, most retinas showed three relatively isolated blebs and the injected solution may diffuse within the bleb after injection. If the injected solution with green dye only occupied one bleb, we determine that the coverage is less than 50% (Fig 2G); if the green dye occupied two blebs, we determine that the coverage is 50–70% (Fig 2H); if all blebs were filled with injected green solution, we determine that the coverage is 80–100% (Fig 2I). From our experience, to reach a long-term (5–12 months) rescue in a mouse model with slow retinal degeneration, it is necessary to have at least 50% (two blebs) of the retina treated, or the rescue effect will diminish in short-term; for a mouse model with early onset/rapid retinal degeneration, it is necessary to have more than 80% (all blebs) of the retina treated to avoid the fading of restored function. That is one of the reasons why we excluded the eyes with less than 50% coverage from the study. In fact, most of the eyes with less than 50% coverage are the results of failed procedures, usually acompanied by many severe complications, including massive hemorrhage of iris and retina, severe traumatic cataract, severe corneal opacity, and severe cornea-iris adhesion. These complications can block the measurement of OCT/fundus and influence ERG results, which is another reason why we have to exclude the eyes with less than 50% coverage from the study.

Our previous studies have suggested that using gene therapy techniques by trans-corneal subretinal injection of vectors containing therapeutic genes could restore visual function in retinal degenerative mice [2–5,7,20,24]. Due to technical difficulties and procedure variations, the existing time of subretinal injection-induced RD/bleb filled with injected solution and its effect on mouse morphology and function are unclear. Therefore, it is necessary to introduce a comprehensive surgical approach for subretinal injection and verification of the duration by a non-invasive OCT machine, which can show in real time the retinal structure, as well as the functional and histological alterations following injection.

Different from the report by Nour et al. that all detached retinas reattached within 24 h post-injection in mouse eyes [25], our study, using OCT, showed that minor retinal detachments could still be observed in many injected eyes, especially in the group with 80–100% coverage at post-injection day 1, although most of the retinas reattached by post-injection day 2. This discrepancy may have arisen from the fact that we only chose eyes with at least 50% of the retina detached and filled with injected solution. We also noticed that although 100% of the retina could automatically detach immediately after procedure in some eyes from the pseudo-injection group, most parts of retina reattached with very limited RD at post-injection day 1. Additionally, almost all retinas reattached completely at post-injection day 2, which is faster than those in the injected groups. This might be because injected solution and/or bigger blebs need more time for absorption.

The b-wave amplitudes of combined ERGs declined differently in each experimental group at 5 weeks post-injection. In most cases of successful injections, the scar in the cornea is very dilute and is hard to see a couple of months after injections. Furthermore, as we indicated in the methods, the pupil is fully dilated and an incision was made in the cornea near the limbus but within pupil range. That means the scar is not in the center of the cornea and is not in the normal pupil area. This is the reason why successfully treated mouse models can see very well in our previous studies [3,5,7]. In some cases of successful injections, mild corneal opacity, mild cornea-iris adhesion, limited cataract due to injection, or mild iris hemorrhage can occur. These damages of anterior segments may be the main reasons why there is 7% ERG reduction in the pseudo-injection group. There is no statistical difference between the b-wave amplitudes of uninjected eyes and pseudo-injected eyes. This means the injection-related damages from anterior segments is not significant, which is very important for the trans-corneal route of the subretinal injection methods.

The declined amplitudes in the injected groups had statistical differences compared with those in the uninjected group. This may be related to the fact that a larger RD filled with injected solution may lead to longer RD persistence and more photoreceptor/outer segment damages, because most of the retinal functional proteins synthesis occurs in the photoreceptor outer segment [26]. These may be the reasons why there is more ERG reduction in 80–100% coverage group (22%) compared to 50–70% group (16%) and pseudo-injection group (7%). The 22% ERG reduction in 80–100% coverage eyes should be caused by injection-related damages from both anterior segments (about 7%) and retina (15%).

This study describes the detailed surgical process of trans-corneal subretinal injection, which is suitable for mice aged P14 or older, when the eyes are open. In fact, in mice younger than P14, especially within 1 week after birth, trans-corneal subretinal injection could severely damage the cornea, iris, lens, and retina because of the smaller eyeballs, underdeveloped cornea and lens, and difficulty in achieving mydriasis. In such cases, trans-scleral subretinal injection should be chosen [16]. A Previous study has suggested that injection-related injury on the retinal structure and function caused by trans-corneal subretinal injection disappeared within 5 weeks [12]. Although we found the injection-related changes in morphology and function are small in the present study, although we could still observe shortened photoreceptor outer segments in the areas close to the injection site at 5 weeks post-injection. Further scrutiny of different time points after injection may reveal specific changes in the retinal structure and function. The effect on the RPE cells also needs further investigation. Trans-corneal subretinal injection is a method that is safe, effective, and practical for P14 or older mice. Nevertheless, various surgical complications to ocular tissues can result, especially by an untrained ‘new hand’. These complications include trauma to cornea, iris, lens, and the retina [21]. A detailed protocol as we described here, with practice, can reduce such injection-related complications. The following precautions should be followed for an effective injection. First, full mydriasis before injection should be ensured [7]. For cornea puncture, the punctured point should not be too close to the edge of the pupil as the pupil may constrict when the cornea is touched with a needle. This would result in injury to the iris before the needle can reach the retinal surface through the anterior chamber. Inadequate mydriasis is also the main cause of iris bleeding during the procedure and iris adhesion post-injection. Second, the 30 ½-gague disposable needle should make only a shallow vertical puncture (and not fully penetrate) initially. To avoid rapid leaking of the aqueous humor and touching the lens, the orientation of the needle should be changed to make it parallel to the lens before penetrating the corneal layers and entering the anterior chamber. Third, to prevent post-surgical iris adhesion, full pre-surgical and post-surgical mydriasis using atropine eye ointment is important.

We used to try 1 or ½ inch of 32g, 33g and 35g needles for subretinal injection in our previous studies of AAV-mediated gene therapy. As we indicated in the manuscript, the most important risk factor for this trans-corneal subretinal injection is pupil dilation, which shows individual differences in each mouse. Although it is difficult to compare the 33g with the 32g or 35g needle while maintaining the other more important risk factors unchangeable in rescue project, we found 33g ½ inch needle is easy to get better rescue effect with our procedure [2–7]. It is also true that our current procedures were summarized from the injection of P14 or older mice with viral vector suspensions, but not cells or tissues. This makes it necessary to adjust the injection settings, including needle size/volume, for either neonatal mice or cell/small tissue injections [27], which is not the target of this study.

In summary, we have provided in detail, a safe and effective method of trans-corneal subretinal injection, which is widely used in ophthalmic research [2–5,7,20,24]. We have also evaluated the changes in morphology and function of the eye at 5 weeks post-injection. Our detailed protocols should be useful for the performance of subretinal injection.
